# Body weight variation is not an independent factor in the determination of functional hypothalamic amenorrhea in anorexia nervosa

**DOI:** 10.1007/s40618-023-02207-z

**Published:** 2023-10-09

**Authors:** C. Cacciatore, B. Cangiano, E. Carbone, S. Spagnoli, M. P. Cid Ramirez, N. Polli, M. Bonomi, L. Persani

**Affiliations:** 1 Department of Endocrine and Metabolic Diseases, IRCCS Istituto Auxologico Italiano, Piazzale Brescia 20, 20149 Milan, Italy; 2 Department of Medical Biotechnologies and Translational Medicine, University of Milan, 20100 Milan, Italy; 3 Secciòn Endocrinologia y Diabetes, Hospital Clìnico Universidad del Chile, Santiago, Chile

**Keywords:** Amenorrhea, Body mass index, Thyroid hormone, Leptin, Eating disorder, Tri-iodothyronine

## Abstract

**Objective:**

Functional hypothalamic amenorrhea (FHA) is one of the foremost manifestations in anorexia nervosa (AN), but a subset of patients have menses despite marked weight loss and underweight. The aim of our study was to investigate parameters potentially influencing FHA in AN.

**Design and methods:**

In this observational retrospective study, we selected 114 female patients with AN who completed a 12 months semi-residential rehabilitation program and a subsequent 12 months outpatient follow-up. We divided our sample into three groups: “Group 0” patients who experienced FHA and recovered their menses, “Group 1” persistent FHA, “Group 2” never experienced FHA, and looked for clinical and hormonal correlations.

**Results:**

At the enrollment, the BMI was higher in Group 2 than in Group 1 (*p* = 0.0202), but the last follow-up weight was higher in Group 1 (*p* < 0.0001) despite persistent amenorrhea. At logistic regression, the higher BMI at which patients experienced amenorrhea was the main prediction factor for persistent FHA. Notwithstanding comparable leptin levels at admission, they improved significantly at discharge only in Groups 0 and 2 (*p* = 0.0054 and *p* = 0.0104, respectively). FT3 at admission was significantly higher in Group 2 than in Group 0 (*p* = 0.0249).

**Conclusions:**

FHA does not correlate strictly with body weight variations in AN patients, indicating a multifactorial origin, likely including an individual predisposition. Higher FT3 levels identify patients who continue having menses at extremely low BMI. AN patients with persistent FHA constitute a subgroup in whom estroprogestins should be considered after significant weight recovery to prevent prolonged tissue hypoestrogenism.

**Supplementary Information:**

The online version contains supplementary material available at 10.1007/s40618-023-02207-z.

## Introduction

According to DMS-5 criteria, anorexia nervosa (AN) is characterized by persistent restriction in caloric intake, intense fear of gaining weight or behavior that interferes with weight gain, bodily disrespect that affects self-esteem levels or non-recognition of the severity of the underweight condition [1].

One of the most frequent clinical complications of AN is functional hypothalamic amenorrhea (FHA), a form of chronic anovulation without organic causes but due to multiple interacting factors [2–4].

Amenorrhea and anorexia are so closely related that in DSM-IV, this complication had been included among the diagnostic criteria of AN; in DSM-V, however, it has been excluded because it cannot be evaluated in some categories of patients such as pre-pubertal patients or patients with delayed puberty, menopausal women, those taking oral contraceptives and male patients [5].

In AN, FHA is the result of an energy deficit due to malnutrition, physical hyperactivity or both; these conditions can induce alterations in metabolic and reproductive hormones [6]. During a chronic caloric restriction, adaptive mechanisms act to diminish basal metabolic rate and preserve calories for vital functions. In such condition, GnRH pulsatility is decreased and LH secretion becomes similar to that of pre-pubertal females with a diminsihed pulse amplitude and frequency, mainly during the day hours [7, 8].

The leptin decrease is considered one of the major signal dysfunctions leading to FHA. A positive correlation between leptin and LH secretion was indeed demonstrated and the increase in circulating leptin concentrations > 1.85 ng/mL was reported to be associated with menstrual recovery [9, 10]. However, the persistence of amenorrhea is not uncommon in women who have regained weight, regardless of the normalization of leptin levels [11]. Nevertheless, the restoration of a normal body weight was reported to be the most important clinical parameter for the functional recovery of the hypothalamic–pituitary–gonadal (HPG) axis [12–14], even if the recovery of menses can frequently be delayed by several months [15, 16]. Generally, the resumption of menses is associated with a 90% recovery of the ideal body weight, but a considerable number of patients (about 1/5) was reported to have a prolongation of amenorrhea even after an adequate weight gain [17–21]. High urinary cortisol, physical hyperactivity and a low body fat mass have been found as risk factors for persistent FHA [18–22].

Furthermore, a subgroup of AN patients do not present FHA, but maintain regular menstrual cycles despite a manifest underweight. One previous study described significant differences in fat mass, leptin levels and in the severity of psychological disorder when eumenorrheic or amenorrheic patients were compared [23].

Despite the efforts of several groups, the relation underlying FHA in AN is still largely unexplained. The aim of our study is to investigate the anthropometric, hormonal and psychological parameters in patients with restrictive-type anorexia nervosa to obtain insights into the factors associated with the onset, resolution or persistence of FHA.

## Materials and methods

### Patients

We revised data from 297 patients affected with eating disorders who were part of a hospital rehabilitation program in the Division of Endocrine and Metabolic Diseases of the IRCCS Istituto Auxologico, since 2011 up to 2018. This is a semi-resident rehabilitation program generally proposed for AN patients with a disease history shorter than 2 years. The program is multidisciplinary and includes either medical, psychological or nutritional interventions.

We selected 226 female patients with AN diagnosis based on DMS-5 criteria, who had reached a BMI > 17.5 kg/m^2^ after a rehabilitative intervention of 6–12 months and did not worsen their BMI during a follow-up period of 12 months. Criteria of exclusion were (i) primary amenorrhea, (ii) previous diagnosis of polycystic ovary syndrome (PCOS), (iii) use of all the drugs potentially interfering with the HPG axis (e.g., anti-dopaminergic agents or estroprogestins), and (iv) intense exercise for >5 h a week.

After informed consent, 114 patients were divided into three groups, according to the variable menstrual history: Group 0 (*n* = 74) included AN patients who experienced FHA during the critical illness and then recovered menstrual cyclicity during rehabilitation; Group 1 included 26 AN patients having persistent amenorrhea despite significant weight gain during rehabilitation; Group 2 (*n* = 14) included all eumenorrheic AN patients, who persistently had regular menses throughout the illness period despite a severe weight loss and prominent underweight.

### Detection of anthropometric parameters

Body weight was measured using the WUNDER Rb300 electronic scale. The body height was measured with a HARPENDEN stadiometer and body mass index (BMI) was calculated as weight (kg)/height (m)^2^.

### Biochemical parameters

Blood samples were periodically taken at 8 am after overnight fasting of at least 8 h. Hormone parameters, such as estradiol, gonadotropins and thyroid function tests, were assessed by routine immunoassays (Roche); leptin was assessed by ELISA (DRG Leptin Sandwich ELISA).

### EDI-2 questionnaires

The severity of psychiatric symptoms was evaluated by administering the EDI-2, a 64-item, self-report questionnaire with eight subscales [24] recognized as valid in the identification and characterization of patients with eating disorders. EDI-2 evaluates behavior and attitude related to nutrition, weight and body shape (drive for thinness, bulimia, body dissatisfaction, impulsiveness) and psychological characteristics (such as inadequacy, perfectionism, self-confidence, fear of maturity, asceticism, social insecurity and enteroceptive capacity).

The EDI-Symptom Checklist is used to measure the frequency of manifestations such as binge eating, abuse of laxatives or diet pills, physical hyperactivity and amenorrhea [25].

### Statistical analysis

Quantitative variables were reported as mean and standard deviation (or as median and interquartile range if the variable was not normally distributed). Shapiro–Wilk test was used to evaluate the normal distribution of data. The three groups were compared with one-way ANOVA test, or non-parametric Kruskal–Wallis test in case of non-normality. Comparisons between two groups were explored with Student’s *t* test, or non-parametric Wilcoxon test in case of non-normality. Predictors of menstrual cycle relapse were analyzed with a logistic regression. For this analysis, we considered only two groups: patients who experienced FHA and recovered their menses (Group 0) and patients remaining in persistent amenorrhea (Group 1). Repeated measures model was used to explore differences of estradiol, fT3 and leptin levels in the three groups and between admission and discharge. The strength and direction of the association between continuous variables were evaluated with Spearman correlation coefficient. Comparisons of EDI-2 scores at admission and at discharge for each group were explored with the Wilcoxon signed rank test. Statistical significance was defined as a two-sided *p* value < 0.05. Statistical analysis was performed using R software version 4.3.0.

## Results

### Anthropometric parameters

The three groups of patients did not differ in age at menarche or at disease/amenorrhea onset, and age at which the lowest weight was reached (Table 1).

**Table 1:** Comparisons of the clinical characteristics of the three groups of AN patients

Variable	Group 0 (*N* = 74)	Group 1 (*N* = 26)	Group 2 (*N* = 14)	*P* value
Age at menarche (years) median [range IQ]	12 [11–13]	12.5 [12–14]	13 [12, 13]	0.1807
N. missing	0	0	1	
Age at disease onset (years) median [range IQ]	15 [14–17]	16 [14–20]	16 [14–17]	0.8514
Age at amenorrhea onset (years) median [range IQ]	15 [15–17]	15 [14–18]	–	0.9442
N. missing	1	1	14	
Age at lowest body weight (years) median [range IQ]	17 [15–18]	18.5 [15–21]	18 [17–22]	0.1671
N. missing	1	0	1	
Premorbid BMI (kg/m^2^) median [range IQ]	20.6 [18.4–22.5]	20.3 [18.9–22.3]	20.05 [18.2–22.3]	0.9644
Maximum weight loss (kg) median [range IQ]	14.45 [10–18.8]	17.8 [13–20]	12.5 [9.2–17]	0.0730
Enrollment BMI (kg/m^2^) median [range IQ]	15.4 [14.4–16.6]	14.7 [14–15.2]	16.4 [15.1–17.2]	0.0180
BMI post-rehabilitation (kg/m^2^) median [range IQ]	17.85 [17–18.9]	18.33 [17.85–19.1]	18.04 [17.44–19.2]	0.2772
Lowest BMI (kg/m^2^) median [range IQ]	15.2 [14.2–16.1]	14.35 [13.4–15.1]	15.9 [14.2–16.8]	0.0151
Weight loss during the disease (premorbid BMI — lowest BMI) median [range IQ]	5.35 [3.9–6.9]	6.75 [5–7.2]	4.35 [3.6–7.1]	0.1447
Difference between BMI at amenorrhea onset and lowest BMI median [range IQ]	1.6 [0.45–2.96]	3.3 [1.9–4.4]	–	0.0053
Difference between premorbid BMI and BMI at amenorrhea onset median [range IQ]	3.55 [2.22–5.4]	2.7 [1.4–4.2]	–	0.1151
Disease duration (months) median [range IQ]	20 [14–46]	18.5 [14–27]	23 [8–33]	0.8318

Group 0, resumed menses; Group 1, no menses resumption; Group 2, no amenorrhea. Data are expressed as mean ± SD or median [range IQ]. The three groups were compared with one-way ANOVA test, or non-parametric Kruskal–Wallis test in case of non-normality. Comparisons between two groups were explored with Student’s *t *test or non-parametric Wilcoxon test in case of non-normality

When anthropometric parameters of the patients were evaluated, premorbid BMI, maximum weight loss and post-rehabilitation BMI were also comparable among the three groups (Table 1).

At the enrollment, the BMI was significantly different between groups (*p* value = 0.0180), Group 2 had a higher BMI than Group 1 (*p* value = 0.0202), and Group 0 tended to have a higher BMI than Group 1 (*p* value = 0.0560). A significant weight gain was obtained in all groups of patients at discharge of the rehabilitation program and average body weights were maintained in all three groups up to 1 year of follow-up. Consistently, regular menses were maintained in patients of Group 0 and Group 2 for the 1-year follow-up after discharge, while amenorrhea persisted in the patients of Group 1 all along the study period.

**Fig. 1:**
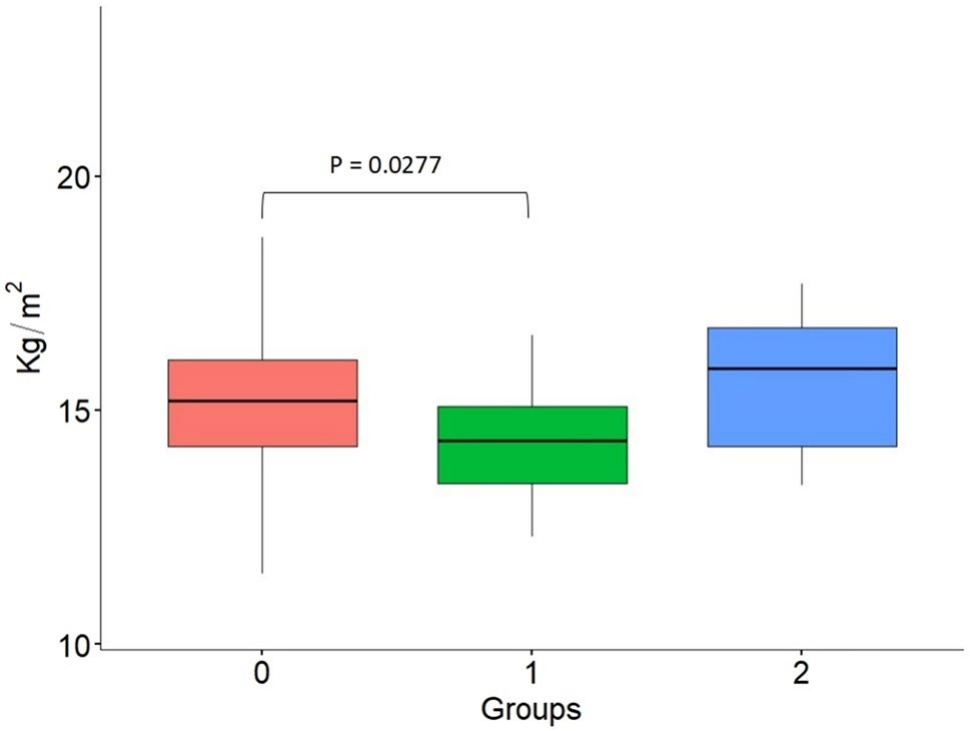
Box plots of the lowest BMI reached during anorexia nervosa (AN) in the three groups: Group 0 (*n* = 74) AN patients who experienced FHA during the critical illness and then recovered menstrual cyclicity during rehabilitation; Group 1 included 26 AN patients remaining in persistent amenorrhea despite significant weight gain during rehabilitation; Group 2 (*n* = 14) included all eumenorrheic AN patients, who persistently had regular menses throughout the illness period

In contrast, the lowest BMI reached by the patients was significantly different among the three groups (*p* value = 0.0151): in Group 1 (persistent amenorrhea), the BMI was significantly lower than in Group 0 (resumed menses) and tended to be lower then in Group 2 (no amenorrhea): 14.38 ± 1.27 versus 15.07 ± 1.58 or 15.95 ± 2.46 (*p* value = 0.0277 and *p* value = 0.0580, respectively) (Fig. 1).

**Fig. 2:**
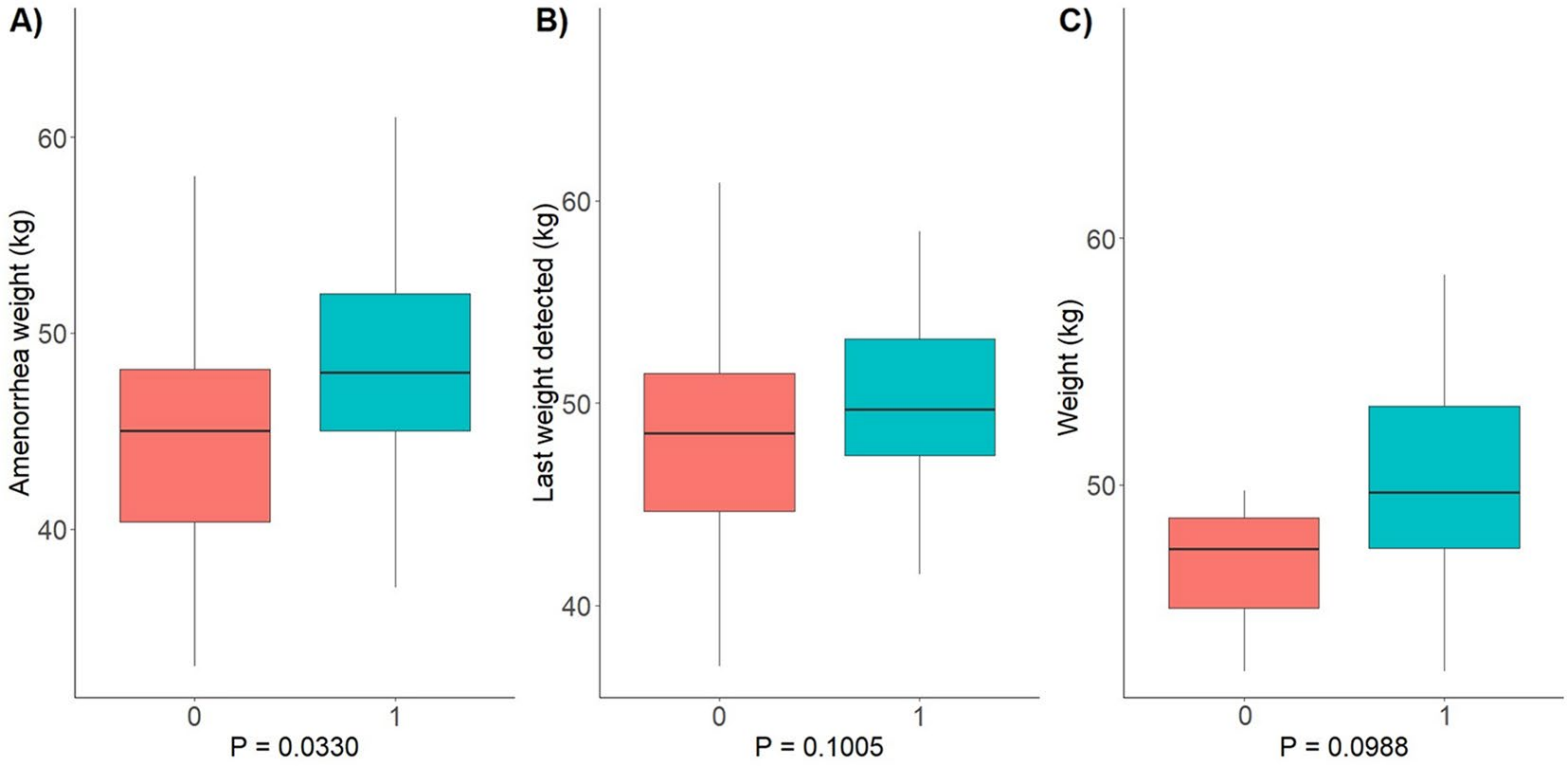
Panel **A** Box plot of the comparison of the weight at which amenorrhea started in Group 0 and Group 1; Panel **B** Box plot of the comparison of the last weight recorded at follow-up in Group 0 and Group 1; Panel **C** Box plot of the comparison of the weight at which Group 0 resumed menses and the last weight detected of Group 1

In addition, we found that the weight at which patients experienced amenorrhea was significantly higher in Group 1 (persistent amenorrhea) than in Group 0 (resumed menses) (48.5 ± 6.82 vs 45.1 ± 5.79 kg, *p* value = 0.0330) (Fig. 2, Panel A). Also, a logistic regression evaluating the association between the persistence of FHA and fT3, enrollment BMI, age at disease onset, lowest BMI and BMI at amenorrhea onset found BMI at amenorrhea to be the only significant association (*p* = 0.05).

The weight measured 12 months after discharge from the rehabilitation was not significantly higher in patients with persistent amenorrhea (50.5 ± 5.76 vs 48.3 ± 5.20 kg; *p* value = 0.1005) if the weight at 12 months follow-up in these patients is compared with the one at which Group 0 regained regular menses (50.5 ± 5.76 vs 46.5 ± 3.29 kg; *p* value = 0.0958) (Fig. 2, Panel B and Panel C).

**Fig. 3:**
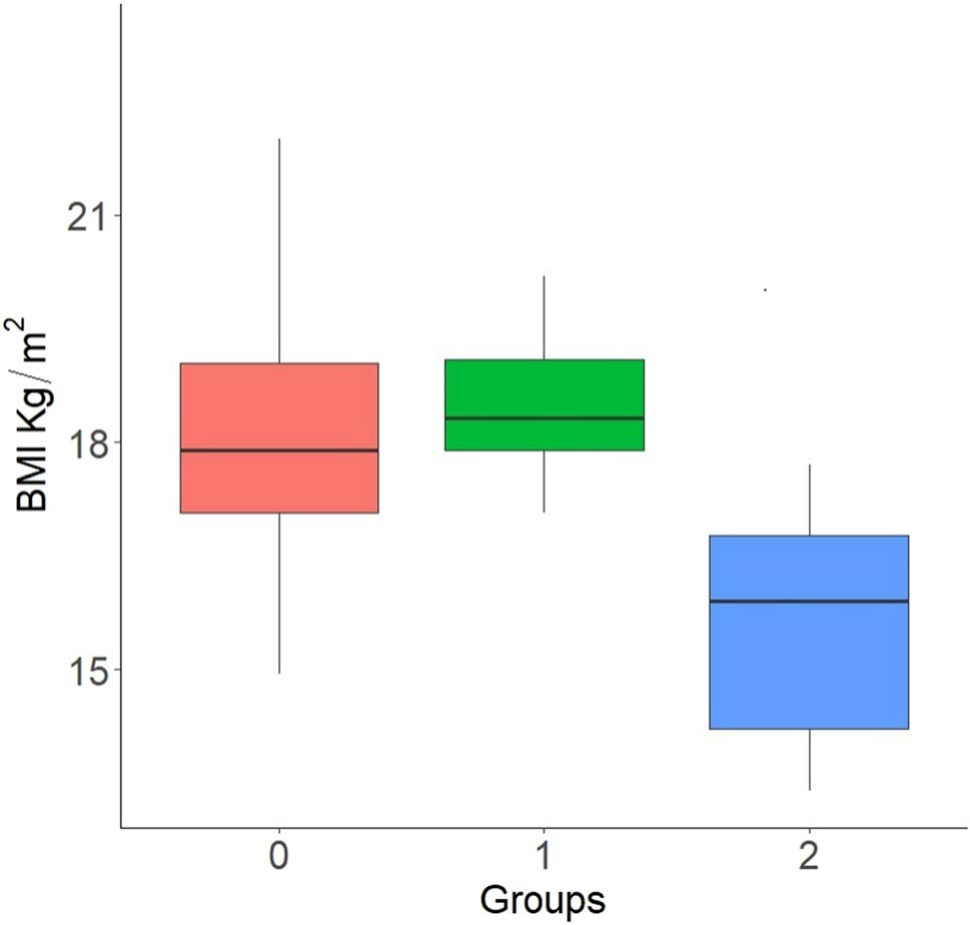
Box plot of BMI comparisons between groups. For AN patients who experienced FHA during the critical illness and then recovered menstrual cyclicity during rehabilitation (group 0), the BMI at which the group resolved amenorrhea was considered, for AN patients remaining in persistent amenorrhea (group 1) the last recorded BMI was considered, and, finally, for eumenorrheic AN patients (group 2) the lowest recorded BMI was considered

As far as comparisons with Group 2 (no amenorrhea) are concerned, the only statistically significant data regard the minimum BMI at which Group 2 patients continued to have their menses, that is, markedly lower than BMI at which Group 0 patients recovered from amenorrhea (Gr.2: 15.95 ± 2.46 vs Gr.0: 18.04 ± 1.63 kg/m^2^, *p* value = 0.0002) and also lower than the last BMI detected during follow-up of Group 1 patients who had persistent amenorrhea (Gr.2: 15.95 ± 2.46 vs Gr.1: 18.7 ± 1.31 kg/m^2^; *p* value < 0.0001) (Fig. 3)).

### Hormonal parameters

As expected, estradiol serum levels were statistically different among the groups of patients who were in amenorrhea and the group of patients who instead had regular menses (*p* value < 0.0001).

**Fig. 4:**
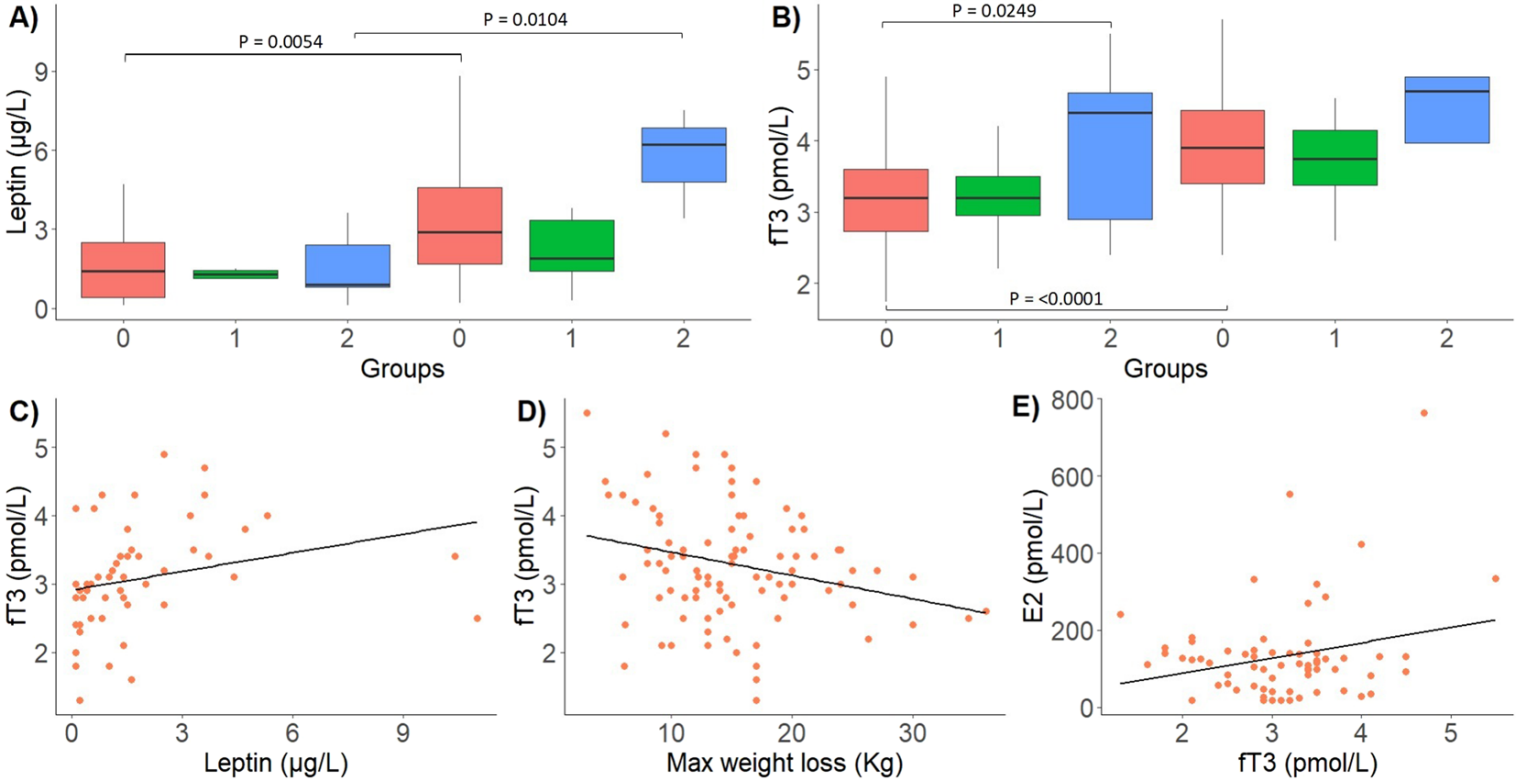
Panel **A** Box plot of the comparison between leptin levels in the three groups and between admission and discharge; panel **B** box plot of the comparison between fT3 levels in the three groups and between admission and discharge; panels **C**–**E** linear regressions: fT3 versus leptin (**C**) and maximum weight loss (**D**); estradiol versus fT3 (**E**)

At admission and at discharge, the circulating leptin levels were similar among the three groups (Gr.0: 1.92 ± 2.36 vs Gr.1: 1.56 ± 1.34 vs Gr.2: 1.56 ± 1.42 μg/mL; *p* value = 0.9689 and *p* value = 0.0885). Leptin values increased from the first control to discharge in Groups 0 (resumed menses) and 2 (no amenorrhea) (*p* value = 0.0054 and *p* value = 0.0104, respectively). Group 1 with persistent amenorrhea did not show a significant increase in circulating leptin (Fig. 4, Panel A).

The serum concentrations of fT3 at admission were significantly higher in Group 2 (no amenorrhea) than in the Group 0 (Gr.2: 3.94 ± 1.11 vs Gr.0: 3.2 ± 0.84 pg/mL; *p* value = 0.0249) and tended to be higher than that in Group 1 (Gr.2: 3.94 ± 1.11 vs Gr.1: 3.23 ± 0.56 pg/mL; *p* value = 0.0609). At discharge, fT3 significantly improved in Groups 0 (*p* value < 0.0001) and the values became similar, but not significant among the three groups (Gr.0: 3.98 ± 0.77 vs Gr.1: 3.7 ± 0.57 vs Gr.2: 4.18 ± 1.28 pg/mL; *p* value = 0.3946) (Fig. 4, Panel B). Proportion of patients with normal/low FT3 levels at admission and at the end of follow-up, for each group are reported in Supplementary Table 4.

A positive correlation was found between fT3 and leptin (*R* = 0.47; *p* value = 0.0005) and a negative correlation between fT3 concentration at admission and the maximum weight loss (*R* = − 0.22: *p* value = 0.0348) (Fig. 4, Panel C and Panel D) showing that lower leptin levels or extreme weight loss corresponded to lower fT3 values. We also found a positive and highly significant correlation between fT3 and estradiol (*R* = 0.53: *p* value < 0.0001) (Fig. 4, Panel E).

### Psychologic symptoms

EDI-2 questionnaires were evaluated both in the comparison between the three groups and within each group between admission and discharge. At admission, the score of all patients was comparable. At discharge, we found a significant improvement of all the items only in patients who recovered regular menses (Group 0). The score partially improved in Group 1, whereas the psychological profile of the patients never experiencing amenorrhea (Group 2) was substantially unchanged. (Supplementary Tables 1, 2 and 3).

## Discussion

In this study, we evaluated the gonadal function of patients suffering from AN by analyzing anthropometric, endocrinological, metabolic and psychological data at the onset of illness, during rehabilitation and after recovery. To our knowledge, this is the first study analyzing clinical and biochemical parameters in the three categories of AN patients with distinct menstrual histories: (i) those with FHA resolving during a stable improvement of the disease conditions, (ii) those with protracted amenorrhea despite BMI and psychological recovery or (iii) persistence of regular menses throughout the course of the disease.

The inclusion and exclusion criteria allowed the selection of patients in whom FHA was apparently related to the critical phase of the AN. It is known that weight and BMI are relevant factors influencing the function of the gonadal axis [13, 25, 26]. However, in our cohort, premorbid BMI, age of disease onset or amount of weight loss was similar among the three groups of patients with variable menstrual history, and in contrast with a previous study which defined premorbid BMI as the main predictor for longer duration of amenorrhea [27]. In addition, despite the patients with persistent amenorrhea experienced the lowest BMI values when compared with the other two groups, they were also the ones to enter FHA at higher BMI values. Indeed, also the logistic regression studying together the factors associated with persistence of FHA found the BMI at which patients experience amenorrhea to be the strongest predictors of the recurrence of menses. This finding, together with the lack of significance of the overall weight loss, points to a higher contribution of the individual predisposition to HPG axis disruption than the effect of the severity of the disease. Accordingly, the last weight at follow-up was statistically higher in patients with persistent amenorrhea even when compared with the weight at which the patients of Group 0 resolved the amenorrhea, thus supporting the thesis that weight is not an independent parameter in determining FHA. Even when we compared Group 2 of the eumenorrheic patients with the other two groups, we found that the weight was not a determining factor for the functionality of the HPG axis. In fact, the lowest BMI at which these patients maintained the menstrual cycle is statistically lower compared to the others.

As far as hormonal parameters are concerned, it is known that leptin correlates with patient's weight and nutritional status and with the amount of fat mass [28–32]. Some authors have questioned such correlations: Miller and colleagues [33] reported hypoleptinemia as independent of fat mass in women with FHA. In our study, we found similar leptin levels at admission, with average values below the lower limit of the healthy population (< 3.6 μg/mL). Leptin concentrations statistically improved at the last follow-up visit only in patients who were menstruating at discharge (Groups 0 and 2). In contrast, the increase of leptin levels was limited and not significant in subjects with persistent amenorrhea (Group 1). Low leptin can account for the decreased pulsatility of LH [30], and the lack of its significant increase justifies the persistence of FHA in Group 1; in this group, low leptin concentrations despite weight recovery are indicative of a different body composition, perhaps due to an unbalanced diet with a low fat content, as previously suggested [33].

Interestingly, the average fT3 values at admission were above the lower reference limit (> 3.7 pmol/L) in eumenorrheic patients (Group 2) and statistically higher (*p* value = 0.0249) than in the group of patients recovering from amenorrhea (Group 0), despite similar clinical characteristics at admission (Table 1), and tended to be higher than in the group of patients with persistent amenorrhea (Group 1). At the end of rehabilitation, there was a general improvement in fT3 concentration, but they remained below or at the lower limit of the normal range in a consistent number of patients with permanent amenorrhea (Group 1), thus indicating the persistence of a hypothalamic dysfunction despite the increase of body weight which might result from the lack of a significant leptin rise.

Reinehr et al. [34] hypothesized that leptin could be the link between BMI and fT3; this observation appears partially in disagreement with the results of our study because patients maintaining the gonadal function during the critical phase of the disease (Group 2) had low levels of leptin and normal fT3 at admission; moreover, patients with persistent amenorrhea had an improvement in fT3, but not in leptin levels after rehabilitation. Swenne et al. [35] concluded that fT3 can be used as a nutritional indicator and that the severity and the velocity of weight loss are the strongest predictors of fT3 concentrations. Consistently, we found an inverse correlation of fT3 with the amount of weight loss and a positive correlation between fT3 and leptin levels, as previously described [36]. But in addition, here we observed a highly significant positive correlation between fT3 and estradiol, with fT3 levels tending to an earlier normalization before resumption of menses and rise of circulating estradiol in Group 0, thus indicating fT3 rise as an early marker of the resumption of hypothalamic activity.This adds to the evidence already available for IGF1 [37] as opposed to cortisol levels which are reported to be a predictor of hypothalamic inhibition [38]. Still, the usefulness of fT3 levels in this setting should always be put in context of clinical parameters. In eumenorrheic patients of Group 2 at admission, fT3 levels were significantly higher in association with the conserved gonadal function; this combination may indicate the portion of AN patients with a hypothalamic function that is less sensitive to the effects of caloric restriction and weight loss.

The main limitation of our study is represented by the lack of body composition determinations to support and correlate with the leptin measurements, leaving open the possibility for a relevant role of the psychopathological profile of the patients [39]. Finally, only some endocrine/metabolic parameters have been evaluated, but many others (GH/IGF-1, hypothalamus–pituitary–adrenal axis, other adipokines and gastro-intestinal hormones) may contribute to the HPG axis dysfunction. Eating disorder manifestations, quantified by the EDI-2 questionnaire, were similar among the three groups, both at admission and discharge. However, when we evaluated the improvement of the score within the three groups, we found that only the patients recovering gonadal function (Group 0) showed a significant general improvement of the score involving almost all the categories. The patients with persistent amenorrhea (Group 1) had an intermediate improvement. These results are in partial agreement with those of Miller et al. [40].

In conclusion, to our knowledge this study is the first comparing three different categories of AN patients defined on the basis of menstrual activity. We found that the premorbid weight and the weight loss during AN are not the main determinants of the GnRH neuron activity, but a more important role is played by the minimal BMI reached during the critical phase of the disease. Nevertheless, a significant portion of patients maintain menstrual cycles even at BMI significantly lower than the other two groups, indicating the significant role played by individual factors in the definition of the set-point of hypothalamic dysfunction. This view is reinforced by the findings in patients with persistent amenorrhea (Group 1) showing a body weight higher than in patients resuming their menses, both at the amenorrhea onset and during the follow-up, further supporting the hypothesis that persistent FHA may be a consequence of an intrinsic fragility of the HPG axis, thus supporting the multifactorial origin of the FHA in the AN patients [41, 42].

At admission, fT3 levels are statistically higher in eumenorrheic patients and fT3 variations during rehabilitation often precede the restoration of gonadal function in Group 0, thus indicating fT3 rise as an early indicator of FHA recovery.

### Supplementary Information

Below is the link to the electronic supplementary material.

Supplementary file1 (DOCX 21 KB)

## Data Availability

Access to associated data can be obtained at this link on Zenodo: https://zenodo.org/record/7844752#.ZD-7N85Bw2w. https://doi.org/10.5281/zenodo.7844752.

## References

[1] American Psychiatric Association (2013). Diagnostic and statistical manual of mental disorders (DSM-5).

[2] Gibson D, Workman C, Mehler PS (2019). Medical complications of anorexia nervosa and bulimia nervosa. Psychiatr Clin North Am.

[3] Miller KK (2011). Endocrine dysregulation in anorexia nervosa update. J Clin Endocrinol Metab.

[4] Nagy H, Paul T, Jain E (2022). A clinical overview of anorexia nervosa and overcoming treatment resistance. Avicenna J Med.

[5] Attia E, Becker AE, Bryant-Waugh R (2013). Feeding and eating disorders in DSM-5. Am J Psychiatry.

[6] Allaway HCM, Southmayd EA, De Souza MJ (2016). The physiology of functional hypothalamic amenorrhea associated with energy deficiency in exercising women and in women with anorexia nervosa. Horm Mol Biol Clin Investig.

[7] Boyar RM, Katz J, Finkelstein JW (1974). Anorexia nervosa. Immaturity of the 24-hour luteinizing hormone secretory pattern. N Engl J Med.

[8] Berga SL, Mortola JF, Girton L (1989). Neuroendocrine aberrations in women with functional hypothalamic amenorrhea. J Clin Endocrinol Metab.

[9] Ballauff A, Ziegler A, Emons G (1999). Serum leptin and gonadotropin levels in patients with anorexia nervosa during weight gain. Mol Psychiatry.

[10] Welt CK, Chan JL, Bullen J (2004). Recombinant human leptin in women with hypothalamic amenorrhea. N Engl J Med.

[11] Brambilla F, Monteleone P, Bortolotti F (2003). Persistent amenorrhoea in weight-recovered anorexics: psychological and biological aspects. Psychiatry Res.

[12] Gordon CM (2010). Clinical practice. Functional hypothalamic amenorrhea. N Engl J Med.

[13] Faust JP, Goldschmidt AB, Anderson KE (2013). Resumption of menses in anorexia nervosa during a course of family-based treatment. J Eat Disord.

[14] Gorrell S, Hail L, Reilly EE (2023). Predictors of treatment outcome in eating disorders: a roadmap to inform future research efforts. Curr Psychiatry Rep.

[15] Castellini G, Rossi E, Cassioli E (2020). Predictors of resumption of menses in anorexia nervosa: a 4-year longitudinal study. Psychosom Med.

[16] Föcker M, Bühren K, Timmesfeld N (2015). The relationship between premorbid body weight and weight at referral, at discharge and at 1-year follow-up in anorexia nervosa. Eur Child Adolesc Psychiatry.

[17] Westmoreland P, Krantz MJ, Mehler PS (2016). Medical complications of anorexia nervosa and bulimia. Am J Med.

[18] Jacoangeli F, Masala S, Mezzasalma FS (2006). Amenorrhea after weight recover in anorexia nervosa: role of body composition and endocrine abnormalities. Eat Weight Disord.

[19] Ackerman KE, Slusarz K, Guereca G (2012). Higher ghrelin and lower leptin secretion are associated with lower LH secretion in young amenorrheic athletes compared with eumenorrheic athletes and controls. Am J Physiol Endocrinol Metab.

[20] Christo K, Cord J, Mendes N (2008). Acylated ghrelin and leptin in adolescent athletes with amenorrhea, eumenorrheic athletes and controls: a cross-sectional study. Clin Endocrinol (Oxf).

[21] Russell M, Stark J, Nayak S (2009). Peptide YY in adolescent athletes with amenorrhea, eumenorrheic athletes and non-athletic controls. Bone.

[22] Karountzos V, Lambrinoudaki I, Tsitsika A, Deligeoroglou E (2017). The role of total body fat mass and trunk fat mass, combined with other endocrine factors, in menstrual recovery and psychopathology of adolescents with anorexia nervosa. Gynecol Endocrinol.

[23] Traboulsi S, Itani L, Tannir H (2019). Is body fat percentage a good predictor of menstrual recovery in females with anorexia nervosa after weight restoration? A systematic review and exploratory and selective meta analysis. J Popul Ther Clin Pharmacol.

[24] Garner D, Olmstead M, Polivy J (1983). Development and validation of a multidimensional eating disorders inventory for anorexia nervosa and bulimia. Int J Eat Disord.

[25] Golden NH, Jacobson MS, Schebendach J (1997). Resumption of menses in anorexia nervosa. Arch Pediatr Adolesc Med.

[26] Swenne I (2004). Weight requirements for return of menstruations in teenage girls with eating disorders, weight loss and secondary amenorrhoea. Acta Paediatr.

[27] Dempfle A, Herpertz-Dahlmann B, Timmesfeld N (2013). Predictors of the resumption of menses in adolescent anorexia nervosa. BMC Psychiatry.

[28] Grinspoon S, Miller K, Coyle C (1999). Severity of osteopenia in estrogen-deficient women with anorexia nervosa and hypothalamic amenorrhea. J Clin Endocrinol Metab.

[29] Ferron F, Considine RV, Peino R (1997). Serum leptin concentrations in patients with anorexia nervosa, bulimia nervosa and non-specific eating disorders correlate with the body mass index but are independent of the respective disease. Clin Endocrinol (Oxf).

[30] Hebebrand J, Muller TD, Holtkamp K, Herpertz-Dahlmann B (2007). The role of leptin in anorexia nervosa: clinical implications. Mol Psychiatry.

[31] Baskaran C, Eddy KT, Miller KK (2016). Leptin secretory dynamics and associated disordered eating psychopathology across the weight spectrum. Eur J Endocrinol.

[32] Anusha K, Hettiaratchi UPK, Athiththan LV, Perera PPR (2019). Inter-relationship of serum leptin levels with selected anthropometric parameters among a non-diabetic population: a cross-sectional study. Eat Weight Disord.

[33] Miller KK, Parulekar MS, Schoenfeld E (1998). Decreased leptin levels in normal weight women with hypothalamic amenorrhea: the effects of body composition and nutritional intake. J Clin Endocrinol Metab.

[34] Reinehr T, Isa A, De Sousa G (2008). Thyroid hormones and their relation to weight status. Horm Res.

[35] Swenne I, Stridsberg M, Thurfjell B, Rosling A (2009). Triiodothyronine is an indicator of nutritional status in adolescent girls with eating disorders. Horm Res.

[36] Onur S, Haas V, Bosy-Westphal A (2005). L-tri-iodothyronine is a major determinant of resting energy expenditure in underweight patients with anorexia nervosa and during weight gain. Eur J Endocrinol.

[37] Cominato L, Da Silva MMX, Steinmetz L (2014). Menstrual cycle recovery in patients with anorexia nervosa: the importance of insulin-like growth factor 1. Horm Res Paediatr.

[38] Arimura C, Nozaki T, Takakura S (2010). Predictors of menstrual resumption by patients with anorexia nervosa. Eat Weight Disord.

[39] Saladino CF (2014). The efficacy of Bioelectrical Impedance Analysis (BIA) in monitoring body composition changes during treatment of restrictive eating disorder patients. J Eat Disord.

[40] Miller KK, Grinspoon S, Gleysteen S (2004). Preservation of neuroendocrine control of reproductive function despite severe undernutrition. J Clin Endocrinol Metab.

[41] Caronia LM, Martin C, Welt CK (2011). A genetic basis for functional hypothalamic amenorrhea. N Engl J Med.

[42] Hietamäki J, Hero M, Holopainen E (2017). GnRH receptor gene mutations in adolescents and young adults presenting with signs of partial gonadotropin deficiency. PLoS ONE.

