# Supplementary material for: Body weight variation is not an independent factor in the determination of functional hypothalamic amenorrhea in anorexia nervosa
Source: J Endocrinol Invest. 2023 Oct 9;47(4):903–11. doi: 10.1007/s40618-023-02207-z (PMC10965633; doi:10.1007/s40618-023-02207-z)
Supplement: Supplementary file 1 — Supplementary file1 (DOCX 21 KB) [file 40618_2023_2207_MOESM1_ESM.docx]

**Supplementary Table 1** Comparison of EDI-2 scores at Group 0 admission and discharge. Values are reported as mean ± standard deviation.

| **EDI-2 questionnaires** | **Admission** | **Discharge** | **P-Value** |
| --- | --- | --- | --- |
| Impulse to Thinness | 13.49 ± 7.21 | 4.45 ± 5.8 | <.0001 |
| Bulimia | 1.37 ± 2.43 | 0.8 ± 2 | 0.0917 |
| Body dissatisfaction | 13.71 ± 6.29 | 8.24 ± 6.31 | <.0001 |
| Inadequacy | 12.33 ± 7.48 | 7.92 ± 6.44 | <.0001 |
| Perfectionism | 5.42 ± 3.64 | 4.18 ± 3.33 | 0.0061 |
| Interpersonal distrust | 8.56 ± 4.49 | 5.18 ± 4.16 | <.0001 |
| Fear of Maturity | 8.24 ± 5.72 | 5.06 ± 4.1 | <.0001 |
| Asceticism | 7.02 ± 4.66 | 4.77 ± 4.37 | 0.0005 |
| Impulsiveness | 6.34 ± 5.9 | 3.01 ± 4.25 | <.0001 |
| Social Insecurity | 9.55 ± 4.87 | 6.86 ± 4.88 | 0.0009 |
| Enteroceptive capacity | 11.23 ± 7.54 | 4.68 ± 5.88 | <.0001 |

**Supplementary table 2** Comparison of EDI-2 scores at Group 1 admission and discharge. Values are reported as mean ± standard deviation.

| **EDI-2 questionnaires** | **Admission** | **Discharge** | **P-Value** |
| --- | --- | --- | --- |
| Impulse to Thinness | 10.13 ± 8.30 | 3.15 ± 5.1 | 0.0049 |
| Bulimia | 1.39 ± 2.65 | 0.7 ± 2 | 0.5625 |
| Body dissatisfaction | 12.17 ± 5.56 | 8.2 ± 5.75 | 0.0239 |
| Inadequacy | 10.78 ± 7.34 | 6.85 ± 3.97 | 0.0062 |
| Perfectionism | 5.08 ± 4.32 | 2.90 ± 3.4 | 0.0713 |
| Interpersonal distrust | 7.56 ± 5.65 | 5.75 ± 3.81 | 0.132 |
| Fear of Maturity | 6.87 ± 4.31 | 4.9 ± 2.19 | 0.0362 |
| Asceticism | 6.9 ± 5.32 | 3.15 ± 3.64 | 0.0114 |
| Impulsiveness | 6.6 ± 7.27 | 2.35 ± 3.93 | 0.0005 |
| Social Insecurity | 9.34 ± 6.13 | 6.55 ± 3.85 | 0.0112 |
| Enteroceptive capacity | 10.04 ± 8.23 | 4.70 ± 6.89 | 0.0142 |

**Supplementary Table 3** Comparison of EDI-2 scores at Group 2 admission and discharge. Values are reported as mean ± standard deviation.

| **EDI-2 questionnaires** | **Admission** | **Discharge** | **P-Value** |
| --- | --- | --- | --- |
| Impulse to Thinness | 10.37 ± 8.46 | 3.42 ± 5.35 | 0.375 |
| Bulimia | 0.75 ± 1.03 | 0 ± 0 | 0.25 |
| Body dissatisfaction | 14.62 ± 8.89 | 7.14 ± 7.92 | 0.4063 |
| Inadequacy | 9.75 ± 7.24 | 6.28 ± 4.85 | 0.125 |
| Perfectionism | 2.87 ± 2.74 | 2.85 ± 2.79 | 0.625 |
| Interpersonal distrust | 7.5 ± 4.89 | 5.57 ± 4.03 | 0.4688 |
| Fear of Maturity | 8.25 ± 5 | 3.57 ± 2.93 | 0.125 |
| Asceticism | 7.62 ± 4.27 | 4 ± 2.58 | 0.125 |
| Impulsiveness | 6.5 ± 6 | 2.85 ± 4.98 | 0.5625 |
| Social Insecurity | 8 ± 4.87 | 6.71 ± 5.28 | 0.4688 |
| Enteroceptive capacity | 12.88 ± 8.89 | 4.57 ± 5.03 | 0.0938 |

**Supplementary Table 4.** Proportion of patients with normal/low FT3 levels at admission and at the end of follow-up, for each group.

|  |  | **Group 0** | **Group 1** | **Group 2** |
| --- | --- | --- | --- | --- |
| **fT3 admission** | fT3 ≥ 3.7 | 15 | 5 | 6 |
|  | fT3 < 3.7 | 47 | 18 | 4 |
| **fT3 dimission** | fT3 ≥ 3.7 | 31 | 9 | 3 |
|  | fT3 < 3.7 | 13 | 5 | 1 |
